# Comparative analysis of COVID-19 vaccine and booster acceptance among healthcare workers in public and private sectors in KwaZulu-Natal, South Africa

**DOI:** 10.4102/hsag.v31i0.3331

**Published:** 2026-07-07

**Authors:** Viloshini K. Manickum, Lehlohonolo J. Mathibe

**Affiliations:** 1 Discipline of Pharmaceutical Sciences, School of Health Sciences, University of KwaZulu-Natal, Durban, South Africa

**Keywords:** COVID-19 vaccine, booster acceptance, hesitancy, healthcare workers, multifaceted

## Abstract

**Background:**

The coronavirus disease 2019 (COVID-19) pandemic posed unprecedented global health challenges, necessitating accelerated COVID-19 vaccine development. However, vaccine and booster hesitancy among healthcare workers (HCWs) presented a barrier globally and in South Africa (SA).

**Aim:**

This study aimed to determine the level of vaccine and booster acceptance among HCWs in public, private and public-private sectors in KwaZulu-Natal (KZN), SA, and to identify the key reasons of vaccine and booster acceptance and refusal.

**Setting:**

This study was conducted across KZN.

**Methods:**

A quantitative, descriptive, cross-sectional survey was conducted from October 2024 to April 2025 among doctors, nurses and pharmacists using an online questionnaire. Three hundred and thirty-one HCWs participated in this study with a reliable and standardised questionnaire. Data analysis included descriptive and inferential statistics. Relevant ethics committees and participants provided ethical approval.

**Results:**

Average vaccine acceptance rate for vaccine and booster was 92.4% (*n* = 306) and 73.7% (*n* = 244), respectively. Public-private recorded the highest acceptance rates for vaccine (96.3%) and booster (85.1%). Primary reasons for vaccine refusal included vaccine-related illness in family (52.4%, *n* = 11) and rapid development and safety concerns (64%, *n* = 16), predominantly among private sector. Main booster refusal reasons included safety of multiple booster doses (50%, *n* = 32) and antibodies from previous COVID-19 infection, mostly in the public sector.

**Conclusion:**

Addressing complex determinants of vaccine hesitancy across healthcare sectors necessitates an integrated and sustained approach reinforced by multidisciplinary stakeholder engagement.

**Contribution:**

This study contributes to future pandemic preparedness and vaccination programmes by elucidating reasons influencing vaccine acceptance and refusal among HCWs.

## Introduction

During December 2019, in Wuhan City, China, the novel coronavirus disease 2019 (COVID-19) was caused by the severe acute respiratory syndrome coronavirus 2 (Harapan et al. 2020). On 30 January 2020, the World Health Organization (WHO) declared COVID-19 a Public Health Emergency of International Concern (Harapan et al. 2020). Within the subsequent 2 weeks, 49 053 laboratory-confirmed cases and 1381 deaths were reported globally (Harapan et al. 2020). World Health Organization officially declared COVID-19 a global pandemic on 11 March 2020 (WHO 2023). During the first 6 months, the pandemic caused over 1 million deaths worldwide (Hodgson et al. 2021), prompting the accelerated development of COVID-19 vaccines (vaccine/s) (George et al. 2023c) to reduce hospitalisation, disease severity and mortality rates.

By April 2021, South Africa (SA) reported over 1.5 million confirmed COVID-19 infections and about 53 356 fatalities, with KwaZulu-Natal (KZN) contributing 335 177 cases and 10 271 deaths (National Institute for Communicable Diseases [NICD] 2021). To avert more deaths, SA commenced the vaccine rollout with the Sisonke study by vaccinating healthcare workers (HCWs) and expanding vaccines to the general population by the end of 2021 (National Department of Health [NDOH] 2021b). By December 2023, 67% (*n* = 13.6 billion) of the global population received a complete primary series of vaccines, with Africa reporting 33% (*n* = 646.5 million) and SA at 35% (*n* = 41.8 million) (WHO 2024). These low coverage rates among the general population, particularly in Africa and SA, reinforce that vaccine hesitancy constituted a major barrier to the success of vaccination programmes (Ackah et al. 2022). This challenge was further underscored by the considerable variation in HCW vaccine acceptance rates observed globally and in SA, irrespective of their levels of vaccination knowledge (George et al. 2023c; Kigongo et al. 2023; Oberleitner et al. 2022; Verger et al. 2021). Therefore, this reinforced that vaccine hesitancy represents a complex and multifaceted global challenge that transcends national boundaries, socioeconomic statuses and stages of development (Baspakova & Sartayeva 2023).

Healthcare workers such as doctors, nurses and pharmacists, in particular, played a key role during the COVID-19 pandemic, and their knowledge, attitudes and responses to the vaccine and COVID-19 vaccine booster doses (booster doses) offered important insights into general vaccine acceptance and hesitancy (Wang et al. 2022).

Communities and patients depended on HCWs for reliable vaccine information, and HCWs’ views strongly influenced the vaccination choices of patients, families and communities (George et al. 2023c). Unvaccinated HCWs faced a higher risk of COVID-19 infection and hospitalisation (Dror et al. 2020), leading to staff shortages, increased workloads and a reduced ability of the health system to manage the pandemic effectively.

In Israel, the vaccine acceptance rate was reported at 68% and 61% among doctors and nurses, respectively (Dror et al. 2020), whereas the United States reported a higher vaccine acceptance rate of 85.7% (Oberleitner et al. 2022). In France, a complex picture was revealed, with 48.6% of HCWs highly accepting, 23% moderately accepting and 28.4% hesitant (Verger et al. 2021). In sub-Saharan Africa, a systematic review of about 7500 HCWs yielded a vaccine hesitancy rate of 46% (Kigongo et al. 2023). A scoping review conducted by Ackah et al. (2022) in Africa identified that vaccine acceptance rates varied between 6.9% and 97.9% (Ackah et al. 2022).

In SA, the reported vaccine acceptance rates among HCWs varied notably across studies. Adeniyi et al. (2021) found a vaccine acceptance rate of 90.1% (*n* = 1308) in 2021, while a separate study by Wiysonge et al. (2022) reported a substantially lower rate of 50% (*n* = 395) during the same year (Adeniyi et al. 2021; Wiysonge et al. 2022). More recently, George et al. (2023c) documented a vaccine acceptance rate of 89% (*n* = 7763) among HCWs in 2022 (George et al. 2023c). Another SA study (*n* = 89) indicated that only two-thirds of HCWs were supportive of mandatory workplace vaccinations (Naidoo & Taderera 2025). Kigongo et al. (2023) concluded that there was a need to better understand obstacles HCWs faced in vaccinating, as they had the potential to influence the broader public (Kigongo et al. 2023).

Research on the vaccine and booster acceptance rates predominantly concentrated on HCW in high-income countries, with limited data available from the African continent, including SA (George et al. 2023b), and KZN province. While the above studies offer valuable insights into vaccine hesitancy and acceptance, none of these studies specifically compare public sector (PUBS) and private sector (PRIVS) vaccine and booster dose acceptance rates and associated acceptance and refusal reasons. Public sector and PRIVS serve different populations and operate under distinct resource constraints (Wilkinson et al. 2022), which can shape attitudes, access, and behaviours related to vaccination. Therefore, there was a need to identify sector-specific barriers and facilitators and develop targeted interventions to address unique challenges, ultimately improving overall vaccine coverage and public health outcomes.

Furthermore, a lack of studies was noted examining the influence of knowledge, education and workplace training on vaccine and booster acceptance and refusal in PUBS and PRIVS in KZN, SA. Healthcare workers play a central role in vaccine advocacy and public trust (George et al. 2023b), and their acceptance or hesitancy directly impacts community perceptions and vaccination rates.

Therefore, our study aimed to determine the level of vaccine and booster acceptance among PUBS, PRIVS and public-private (PUBPR) HCWs in KZN, SA, and to identify the key drivers of vaccine and booster acceptance and refusal.

The objective of this study was to compare vaccine and booster acceptance rates among PUBS, PRIVS and PUBPR HCWs in KZN, SA and the primary reasons influencing vaccine and booster acceptance and refusal among these sectors.

## Research methods and design

### Study setting

This study was conducted across all 11 Districts in KZN, which is the second largest province in SA. KwaZulu-Natal comprises a combination of urban and rural communities, characterised by diverse socioeconomic backgrounds (Stats SA 2024).

### Study design

This quantitative, descriptive, cross-sectional survey was conducted from October 2024 to mid-April 2025.

A cross-sectional quantitative study design was chosen because it enabled the assessment of COVID-19 vaccine and booster acceptance rates and reasons affecting uptake and refusal at a specific point in time. This approach allowed the collection of standardised data from the study population, facilitating the identification of key demographic and other reasons associated with vaccine hesitancy and acceptance.

### Study population and sample

This study used a purposive and snowballing approach. Purposive sampling was used to specifically target doctors, nurses and pharmacists, ensuring that participants were relevant to the research question. Snowball sampling was incorporated to broaden the participant pool by allowing initial respondents to refer colleagues, which was particularly useful for reaching a population that would have been difficult to access comprehensively through random sampling. The total population of interest in KZN comprised 17 699 professional nurses, 4839 doctors and 1236 pharmacists (Matseke 2023). A sample size of 379 participants was required to detect a statistically significant difference at a 95% confidence interval (*p* < 0.05), with a population proportion of 50% and a 5% margin of error (Calculator.net 2008).

A total of 379 HCWs participated and completed the study questionnaire; however, only 331 met the inclusion criteria. Approximately 13% (*n* = 48) of participants were excluded from the analysis because of not being from KZN, SA. The survey was distributed electronically through WhatsApp and email groups; therefore, it was not possible to determine an exact response rate. Nevertheless, the number of responses obtained surpassed the minimum required sample size (*n* = 379) to ensure adequate statistical power.

Probability sampling was not feasible in this study because of the challenges with accessing a comprehensive centralised registry with contact details, making it impractical to randomly select participants. These limitations necessitated the use of non-probability sampling methods, such as purposive and snowball sampling, to effectively reach and recruit eligible participants.

The use of purposive and snowball sampling in this study introduces potential sampling bias, which may affect the generalisability of the findings. Because participants were recruited through targeted selection and referrals rather than random sampling, the sample may not fully represent the broader population of doctors, nurses and pharmacists in KZN. Additionally, the reliance on electronic distribution methods, such as WhatsApp and email, may have excluded individuals without regular access to these platforms, further influencing the participant pool.

### Inclusion criteria

Inclusion criteria consisted of registered doctors, professional nurses and pharmacists who provided informed consent and were actively practising in KZN. There were no minimum requirements for years of professional experience; however, eligibility was restricted to individuals who were currently practising in PUBS, PRIVS and PUBPR healthcare settings.

### Exclusion criteria

The exclusion criteria encompassed participants not providing informed consent and practising in provinces other than KZN.

### Data collection

A secure, structured Google Form questionnaire was utilised for data collection in this study. Section 1 included background information, followed by Section 2, which contained the informed consent process.

Participants who did not provide consent were automatically exited from the questionnaire upon indicating their decision not to participate in this study. Consenting participants moved to Section 3, which gathered demographic information. Demographic questions included age, gender, profession, race and sector. Section 4 addressed participants’ education and training. Education and training included vaccine understanding, undergraduate and organisational training. Section 5 included chronic disease conditions, COVID-19 infection history and acceptance of the COVID-19 vaccine. Section 6 explored the acceptance of booster doses. All consenting participants completed Sections 2, 3, 4, 5 and 6. Finally, Sections 7 and 8 examined the reasons for refusal of the COVID-19 vaccine and booster doses, respectively. Only participants who refused the vaccine and booster were directed to and completed Sections 7 and 8. The responses were submitted and directly transferred to a securely stored Google Drive spreadsheet.

The study questionnaire Google link was distributed via Gmail and WhatsApp to doctors, nurses and pharmacists in KZN. Participation was voluntary, and both public and PRIVS doctors, nurses and pharmacists were invited to participate. An online survey format was selected due to logistical and practical considerations, facilitating accessibility and ease of participation across different healthcare settings. This approach also enhanced respondent convenience and ensured anonymity, which may have contributed to higher response rates and improved data reliability.

### Data analysis

The data were imported into an Excel™ spreadsheet, and the responses were categorised into PUBS, PRIVS, and PUBPR. SPSS version 29 (IBM, Armonk, NY, United States) was employed to analyse this data. Missing data was noted because of participants not completing some questions. Because this was an online, anonymised survey, the authors were unable to follow up on missing data. Therefore, missing values were disregarded, and analyses were conducted using only completed data. Descriptive statistics were implemented to summarise the demographic information, educational background, training, acceptance of COVID-19 vaccines and boosters, and vaccine refusal of the participants.

Demographic characteristics, including age, profession, sector and race, were defined as independent variables. The Kruskal-Wallis and Mann-Whitney U tests were employed to conduct analysis to evaluate the differences between an independent variable with respect to certain demographic, social and medical variables. These variables included the likelihood of recommending vaccines to friends, participation in organisational training, frequency of question-asking during training, use of chronic medication, vaccine brand received and acceptance of booster doses. The multivariable binomial logistic regression model was used to estimate crude odds ratios and adjusted odds ratios while controlling for potential confounders. The model included variables that demonstrated significant associations in the multivariate analysis (*p* < 0.05) and variables that were considered theoretically important based on previous literature.

### Ethical considerations

Ethical clearance to conduct this study was obtained from the University of KwaZulu-Natal Biomedical Research Ethics Committee (No. BREC 4505/2022) and the KZN Department of Health Ethics Committee (Ref No: KZ_202208_035). Written permission was obtained from the KZN Department of Health and gatekeepers from PRIVS organisations. Participants were fully informed of the study’s purpose and potential benefits and voluntarily agreed to participate. Written informed consent was obtained from all participants before their involvement. There was no risk to participants, as the study did not involve tests or human trials. While there were no direct benefits, the participants contributed to findings that may indirectly enhance the outcomes of future vaccination programmes, particularly during pandemics. Confidentiality of participants’ information was strictly maintained. Questionnaire responses were securely stored on a password-protected Google Drive that was accessible only to the researcher and the study supervisor. In line with institutional research policy, the data will be retained for 5 years.

## Results

The primary outcome of this study was the acceptance of vaccine and booster doses. In addition to this primary outcome, this study also investigated secondary outcomes, including participants’ demographic characteristics (such as age and gender), educational backgrounds, professional roles and perceptions or attitudes towards the acceptance or refusal of the vaccine and booster doses.

### Demographic characteristics

This study examined the demographic characteristics of a total sample of 331 participants, including individuals who both accepted and declined the COVID-19 vaccine and booster doses. However, the number of participants completing the demographic characteristics questions ranged from 327 to 331.

Table 1 indicates that the largest proportion of participants were aged 40–49 years (30.2%) and female (65%), with pharmacists representing the most common professional category (39.2%). Almost half of the participants were PUBS (48.9%) and of Asian/Indian descent (49.5%).

**TABLE 1:** Demographic characteristics of all study participants.

Variable	*n*	%
**Age (years)**		
20–29	19	5.7
30–39	67	20.2
40–49	100	30.2
50–59	82	24.8
60–69	47	14.2
Greater than 70	16	4.8
**Gender**		
Male	114	34.4
Female	214	65.0
Other	1	0.3
**Profession**		
Doctor	108	32.8
Nurse	92	28.0
Pharmacist	129	39.2
**Sector**		
Private sector	138	41.7
Public and private sectors	27	8.2
Public sector	162	48.9
**Race**		
African people	103	31.1
Asian/Indian people	164	49.5
Coloured people	10	3.0
White people	53	16.0

Note: Table 1 shows the demographic characteristics of all participants who met the inclusion criteria. Race, gender and sector: Missing data were noted for 1, 2 and 4 participants, respectively.

### Determinants of COVID-19 vaccine and booster uptake and refusal

#### Vaccine and booster acceptance rate

**FIGURE 1:**
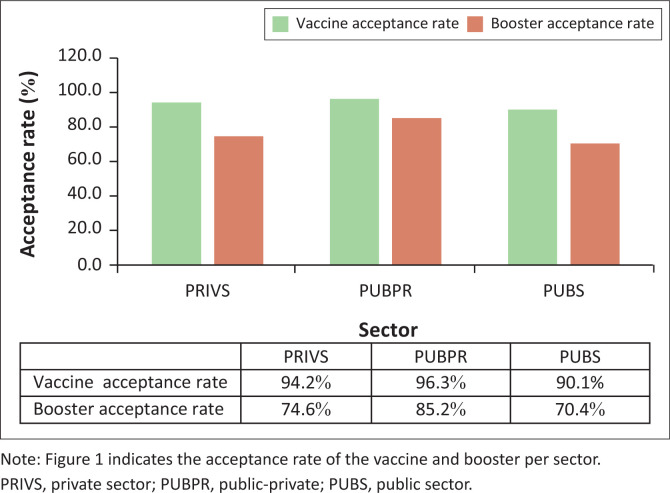
Vaccine and booster acceptance rates versus sector.

The average vaccine acceptance rate for the vaccine and booster in KZN was 92.4% (*n* = 306/331) and 73.7% (*n* = 244/331), respectively. Figure 1 indicates that PUBPR recorded the highest acceptance rates for both the vaccine and the booster dose. Notably, PUBS recorded almost a 4% lower vaccine and booster rate than PRIVS. Janssen Biotech Inc. (J&J) was the commonly received (58.5%, *n* = 80 PUBS; 43%, *n* = 55 PRIVS; 50%, *n* = 13 PUBPR) vaccine.

#### Demographic characteristics of participants accepting vaccine and booster doses

The vaccine was primarily accepted by the 40–49 (32.2%, *n* = 47 PUBS; 30%, *n* = 39 PRIVS) age group. Boosters were received mostly by the 50–59 age group in PUBS (33.3%, *n* = 38) and the 40–49 age group in PRIVS (30.1%, *n* = 31). However, the 50–59 age group was 92.4% (coefficient = –2.571, *p* = 0.001, odds ratio = 0.076) less likely to have received a booster dose than the 20–29-year age group. A statistically significant association (*p* < 0.05) was observed between age and several variables, including the likelihood of recommending vaccines to friends, participation in organisational training, frequency of question-asking during training, use of chronic medication, vaccine brand received, and acceptance of booster doses.

Although females demonstrated a greater acceptance rate of the vaccine (67.6%, *n* = 98 PUBS; 62.8%, *n* = 81 PRIVS; 69.2%, *n* = 18 PUBPR) and boosters (70.2%, *n* = 80 PUBS; 59.8%, *n* = 61 PRIVS, 78.3%, *n* = 18 PUBPR), they were 53.4% (coefficient = –2.571, *p* = 0.001, odds ratio = 0.076) less likely to have taken a booster compared to males. A statistically significant association (*p* < 0.05) was observed between gender and several variables, including participation in organisational training, frequency of question-asking during training, vaccine brand received, perceived increase in protection, as well as pregnancy and breastfeeding status.

Public sector Africans (46.6%, *n* = 68), PRIVS Asians/Indians (62.8%, *n* = 81) and PUBPR Asians/Indians (38.5%, *n* = 10) reported the highest vaccine acceptance rate. Meanwhile, the booster dose acceptance rate was the highest among the PUBS (45.6%, *n* = 52) and PRIVS (65.7%, *n* = 67) Asians or Indians. African participants were 64.6% (coefficient = –1.038, *p* = 0.009, odds ratio = 0.354) less likely to take the booster compared to their white counterparts. Statistical significance (*p* < 0.05) was noted with race and vaccine understanding, use of chronic medication and acceptance of booster doses.

**FIGURE 2:**
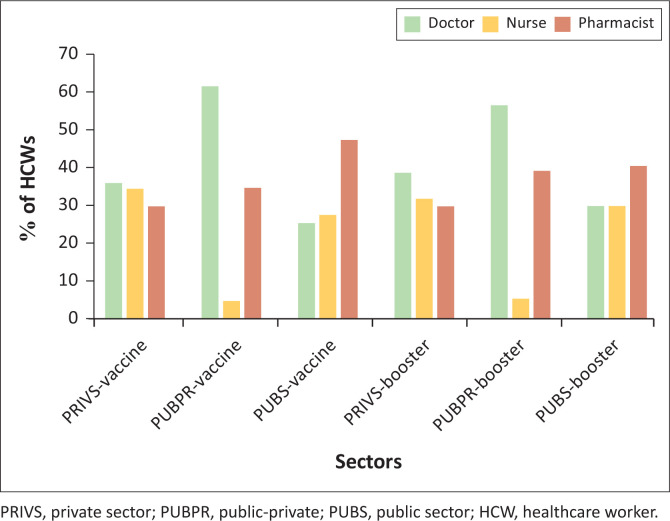
COVID-19 vaccine and booster uptake versus profession.

Figure 2 depicts the acceptance rate of the vaccine and booster versus professions. This study noted that the vaccine acceptance/uptake fluctuated across the different professions, with the highest among PUBPR doctors and PUBS pharmacists (Figure 2). Meanwhile, PRIVS doctors and PUBS nurses recorded a slightly higher uptake of boosters compared to the vaccine. However, almost a 7% decrease in booster acceptance rate was seen with PUBS pharmacists in comparison to the vaccine (Figure 2). A statistically significant relationship (*p* < 0.05) was found between profession and several factors. These factors included vaccine understanding, likelihood of recommending vaccines, participation in training, and types of training attended. Additionally, the frequency of asking questions during training, the vaccine brand received, perceived increase in protection after vaccination, natural immunity to COVID-19, and the presence of COVID-19 symptoms were also significantly (*p* < 0.05) related to profession.

#### Vaccine knowledge

**FIGURE 3:**
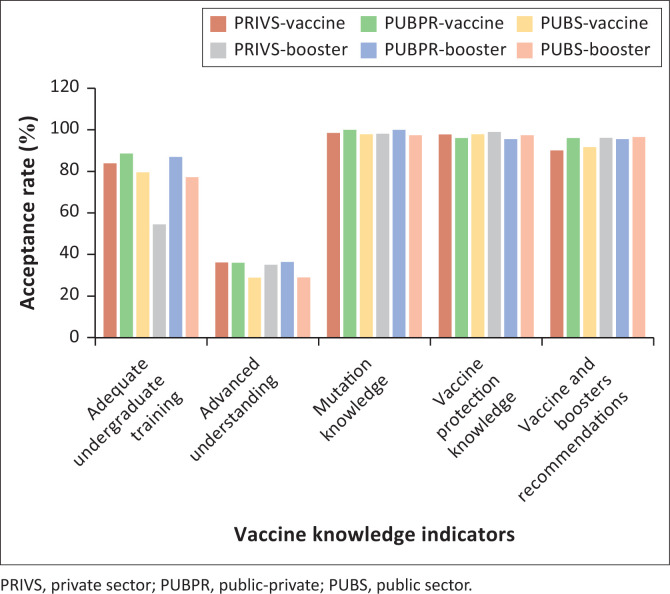
Vaccine knowledge indicators versus acceptance of vaccine and booster doses.

Figure 3 indicates the acceptance rate of vaccines and boosters versus vaccination knowledge indicators per sector. Vaccination knowledge indicators include adequate undergraduate training, advanced understanding, mutations, vaccine protection and recommendations to friends/family. An adequate level of vaccine undergraduate training varied considerably, with PUBPR noting the maximum rate for vaccine (88.5%, *n* = 23) and booster (87%, *n* = 20) and PRIVS booster recipients recording the lowest rate (54.5%, *n* = 87) (Figure 3). A total of 54 participants (20.5%, *n* = 30 PUBS; 16.2%, *n* = 21 PRIVS, 11.5%, *n* = 3 PUBPR) received the vaccine while noting inadequate undergraduate training. Eighteen participants with a lack of undergraduate training declined vaccine (37.5%, *n* = 6 PUBS; 25%, *n* = 2 PRIVS) and booster (14.7%, *n* = 5 PUBS; 17%, *n* = 5 PRIVS).

Figure 3 indicates that almost a third of all participants receiving the vaccine and booster reported an advanced level of understanding of vaccines/immunology. The PUBS noted the lowest rate of advanced understanding for the vaccine (28.8%, *n* = 42 and booster (28.9%, *n* = 33). Altogether 24 participants reported an advanced level of understanding; they still refused vaccine (12.5%, *n* = 2 PUBS; 12.5%, *n* = 1 PRIVS) and booster doses (26.5%, *n* = 9 PUBS; 39.3%, *n* = 11 PRIVS; 33.3%, *n* = 1 PUBPR).

Basic understanding of vaccines was recorded by a total of 22 (87.5%, *n* = 14 PUBS; 87.5%, *n* = 7 PRIVS; 100%, *n* = 1 PUBPR) participants not receiving the vaccine and 44 (73.5% *n* = 25 PUBS; 60.7%, *n* = 17 PRIVS; 66.7%, *n* = 2 PUBPR) not receiving the boosters.

The study found that PUBS, PRIVS and PUBPR recipients of the vaccine and booster showed close to 100% understanding of virus mutations and protective benefits provided by vaccines and boosters.

A greater proportion of PUBS and PRIVS participants recommended the booster to family and friends. A total of 29 participants not receiving vaccine (50%, *n* = 8 PUBS; 100%, *n* = 8 PRIVS; 100%, *n* = 1 PUBPR) and booster (12.1%, *n* = 4 PUBS; 28.6%, *n* = 8 PRIVS) did not encourage uptake among family/friends.

### Workplace training

Workplace training was slightly higher in PUBS for vaccine (75%, *n* = 108) and booster (74.1%, *n* = 83) recipients in comparison to PRIVS. The blended approach was the common training method across all sectors, with a maximum rate noted in PUBS (36.6%, *n* = 53) for vaccines. A total of 26 participants reported not receiving training and subsequently not receiving vaccine (37.5%, *n* = 6 PUBS; 37.5%, *n* = 3 PRIVS, 100%, *n* = 1 PUBPR) and booster (23.5%, *n* = 8 PUBPR; 25%, *n* = 7 PRIVS; 33.3%, *n* = 1 PUBPR).

A total of 21 participants not receiving the vaccine (26.7%, *n* = 4 PUBS; 25%, *n* = 2 PRIVS; 100%, *n* = 1 PUBPR) and booster (15.2%, *n* = 5 PUBS; 28.6%, *n* = 8 PRIVS; 33.3%, *n* = 1 PUBPR) selected non-applicable with respect to the type of training session; so, it remains unclear whether these staff were trained.

About two-thirds of vaccine and booster recipients indicated that they had the opportunity to ask questions.

### Chronic medicine use and coronavirus disease 2019 infection rates

A higher proportion of PRIVS vaccine (45.7%, *n* = 59) and booster (47.1%, *n* = 48) recipients recorded using chronic medicines. Twenty (68.8%, *n* = 11 PUBS; 100%, *n* = 8 PRIVS; 100%, *n* = 1 PUBPR) and 30 (100%, *n* = 3 PUBS; 68.8%, *n* = 11 PRIVS; 60.7%, *n* = 17 PUBPR) participants were not taking chronic medicines and refused the vaccine and booster, respectively.

A higher rate of positive COVID-19 tests was noted in PRIVS for vaccine (59.7%, *n* = 77) and booster (57.8%, *n* = 59). About a third of PUBS, PRIVS and PUBPR participants receiving vaccine and booster recorded having COVID-19 infection once. The majority of PUBS, PRIVS and PUBPR recorded COVID-19 more than five times, accepted the vaccine and booster. Mild and moderate illness was reported by the majority of PUBS, PRIVS and PUBPR participants receiving the vaccine and boosters.

### Reasons for COVID-19 vaccine refusal

Twenty-five participants (9.9%, *n* = 16 PUBS; 5.8%, *n* = 8 PRIVS; 3.7%, *n* = 1 PUBPR) refused the vaccine.

However, the number of participants who provided reasons for each question in Table 2 varied between 21 and 25.

**TABLE 2:** COVID-19 vaccine refusal reasons.

Question	Options	PRIVS		PUBPR		PUBS	
		*n*	%	*n*	%	*n*	%
Family members/friends being ill after taking the vaccine	Agree and strongly agree	5	62.5	0	-	6	37.5
	Disagree and strongly disagree	1	12.5	1	100	3	18.8
	Neutral	2	25.0	0	-	3	18.8
Non-accessibility of vaccines	Agree and strongly agree	0	-	0	-	1	6.3
	Disagree and strongly disagree	4	50.0	1	100	10	62.5
	Neutral	4	50.0	0	-	2	12.5
Use of prescription/chronic medicines	Agree and strongly agree	0	-	0	-	4	25.0
	Disagree and strongly disagree	2	25.0	0	-	5	31.3
	Neutral	6	75.0	0	-	7	43.8
Natural immunity to COVID-19 infection	Agree and strongly agree	3	37.5	0	-	5	38.5
	Disagree and strongly disagree	2	25.0	0	-	3	23.1
	Neutral	3	37.5	1	100	5	38.5
Use of traditional medicines for COVID-19 protection	Agree and strongly agree	1	12.5	0	-	1	6.3
	Disagree and strongly disagree	3	37.5	0	-	9	56.3
	Neutral	5	62.5	1	100	3	18.8
Pregnancy	No	2	25.0	0	-	7	43.8
	Not Applicable	5	62.5	1	100	5	31.3
Breastfeeding	No	2	25.0	0	-	7	43.8
	Not Applicable	4	50.0	1	100	6	37.5
Belief that COVID-19 was fake	Agree and strongly agree	2	25.0	0	-	0	-
	Disagree and strongly disagree	4	50.0	1	100	10	62.5
	Neutral	2	25.0	0	-	6	37.5
Manufacturers increasing profits	Agree and strongly agree	6	75.0	0	-	5	31.3
	Disagree and strongly disagree	0	-	0	-	4	25.0
	Neutral	2	25.0	1	100	7	43.8
Vaccine refusal for religious reasons	Agree and strongly agree	3	37.5	0	-	3	18.8
	Disagree and strongly disagree	4	50.0	0	-	9	56.3
	Neutral	1	12.5	0	-	4	25.0
Absence of faith/trust in the vaccine	Agree and strongly agree	7	87.5	0	-	7	43.8
	Disagree and strongly disagree	0	-	1	100	3	18.8
	Neutral	2	25.0	0	-	6	37.5
Lack of trust in government	Agree and strongly agree	6	37.5	0	-	1	6.3
	Disagree and strongly disagree	0	-	1	100	3	18.8
	Neutral	2	25.0	0	-	12	75.0
Belief that the vaccine had a microchip for tracking/controlling people	Agree and strongly agree	2	12.5	0	-	0	-
	Disagree and strongly disagree	5	31.3	1	100	10	62.5
	Neutral	1	12.5	0	-	6	75.0
The belief that COVID-19 affected fertility and the ability to have children	Agree and strongly agree	3	37.5	0	-	1	12.5
	Disagree and strongly disagree	1	12.5	1	100	9	56.3
	Neutral	4	25.0	0	-	6	75.0
Observing negative effects on social media	Agree and strongly agree	5	62.5	0	-	5	31.3
	Disagree and strongly disagree	1	12.5	1	100	3	18.8
	Neutral	2	25.0	0	-	8	50.0
Antivaxxer influence against vaccination	Agree and strongly agree	2	25.0	0	-	0	-
	Disagree and strongly disagree	4	50.0	1	100	9	56.3
	Neutral	2	25.0	0	-	7	43.8
Quick development of the vaccine and uncertainty of side effects	Agree and strongly agree	6	75.0	0	-	10	62.5
	Disagree and strongly disagree	0	-	1	100	1	6.3
	Neutral	2	25.0	0	-	5	31.3

Note: Table 2 presents the reasons for vaccine refusal. PUBS, PRIVS and PUBPR columns indicate the frequency and percentage of participants who agreed, disagreed or expressed a neutral stance.

COVID-19, coronavirus disease 2019; PRIVS, private sector; PUBPR, public-private; PUBS, public sector.

Table 2 shows that 52.4% (*n* = 11/21) of participants agreed that a family member or friend became ill following vaccine administration, and this was seen in a greater proportion of PRIVS (62.5%, *n* = 5) in comparison to PUBS participants. A total of 68.2% (*n* = 15/22) of participants disagreed that vaccines were not readily available near homes or places of employment. Nearly 54.2% (*n* = 13/24) of the participants reported neutrality and disagreement with their use of prescription or chronic medications, with the highest rate of neutrality in PRIVS. Concerning natural immunity, 40.9% (*n* = 9/22) of participants remained neutral. Meanwhile, 36.4% (*n* = 8/22) agreed about natural immunity, while PUBS (38.5%, *n* = 3) noted a slightly higher rate.

The majority of participants; 52.2% (*n* = 12/23), disagreed with the use of traditional medicine for protection against COVID-19, with this response being most prevalent among PUBS participants (56.3%, *n* = 9). Private sector participants noted a higher rate of agreement (12.5%, *n* = 1) with the use of traditional medicines. Sixty per cent (*n* = 15/25) of all participants disagreed with the belief that COVID-19 was fake. Notably, PRIVS (25%, *n* = 2) only agreed with COVID-19 being fake. Refusal of the vaccine for religious reasons was disagreed with by 54.2% (*n* = 13/24) of participants. Conversely, 25% (*n* = 6/24) agreed that they did not take the vaccine because of religious reasons, with the highest proportion observed in the PRIVS group (37.5%, *n* = 3).

A greater proportion of participants; 44% (*n* = 11/25), agreed with the profit incentive of manufacturers, with the highest observed in PRIVS (75%, *n* = 6). A total of 56% (*n* = 14/25) participants agreed on a lack of faith in the vaccine, with the highest rate observed among the PRIVS (87.5%, *n* = 7). Additionally, 28% (*n* = 7/25) of participants agreed with a lack of trust in the government, with the highest proportion observed in the PRIVS (37.5%, *n* = 6). A total of 64% (*n* = 16/25) of participants disagreed with the vaccine being used as a tracking device, a minority agreed in PRIVS (12.5%, *n* = 2). In contrast, agreement with the presence of a microchip in the vaccine was primarily observed by a minority of PRIVS (12.5%, *n* = 2). A total of 44% (*n* = 11/25) of participants disagreed with vaccines affecting fertility. However, a comparatively higher proportion of PRIVS (37.5%, *n* = 3) agreed with adverse fertility effects.

Equal numbers and proportions (40%, *n* = 10/25) of participants agreed regarding exposure to adverse vaccine effects on social media. Concerning the influence of anti-vaxxer individuals, 56% (*n* = 14/25) of participants disagreed that friends with anti-vaccine views affected their decision-making. Additionally, 8% of participants indicated agreement with antivaxxers influencing their uptake, and this was mostly seen in PRIVS. A total of 64% (*n* = 16/25) of participants agreed that the vaccines were developed rapidly and expressed uncertainty regarding potential side effects. Twenty-eight per cent (*n* = 7/25) of participants reported a neutral stance on this issue, with the highest rate of neutrality observed in the PUBS group (31.3%, *n* = 5).

### Reasons for refusal of COVID-19 vaccine booster doses

The study identified a total of 65 participants (52.3%, *n* = 34 PUBS; 43.1%, *n* = 28 PRIVS; 4.6%, *n* = 3 PUBPR) who refused booster doses. A total of 22 participants did not provide a response to the acceptance of boosters.

Notably, the number of participants who answered follow up questions ranged between 64 and 65, as shown in Table 3.

**TABLE 3:** COVID-19 booster doses refusal reasons.

Question	Options	PRIVS		PUBPR		PUBS	
		*n*	%	*n*	%	*n*	%
Uncertainty about the safety of multiple booster doses	Agree and strongly agree	15	53.6	2	66.7	15	45.5
	Disagree and strongly disagree	5	17.9	1	33.3	3	9.1
	Neutral	8	28.6	0	-	15	45.5
Presence of COVID-19 infection and acquired antibodies	Agree and strongly agree	8	28.6	1	33.3	17	51.5
	Disagree and strongly disagree	7	25.0	0	-	8	24.2
	Neutral	13	46.4	2	66.7	8	24.2
Chronic conditions worsening after taking the vaccine	Agree and Strongly Agree	4	14.3	0	-	6	18.2
	Disagree and strongly disagree	12	42.9	1	33.3	10	30.3
	Neutral	12	42.9	2	66.7	17	51.5
Experiencing side effects after 1st vaccine dose	Agree and strongly agree	16	59.3	1	2.9	17	51.5
	Disagree and strongly disagree	8	29.6	1	33.3	9	33.3
	Neutral	3	11.1	1	33.3	5	15.2
Grade of side effects	Mild	8	28.6	1	33.3	15	45.5
	Moderate	9	32.1	1	33.3	4	12.1
	Severe	2	7.1	0	-	1	3.0
	Not applicable	9	32.1	1	33.3	13	39.4
Reporting of side effects to HCWs	No	11	39.3	2	66.7	14	42.4
	Yes	8	28.6	0	-	5	15.2
	Not applicable	9	32.1	1	33.3	14	42.4
Receiving medicines for side effects	No	8	28.6	0	-	12	35.3
	Yes	12	42.9	2	66.7	8	23.5
	Not applicable	8	28.6	1	33.3	13	38.2
The vaccine was not effective against emerging strains of the virus	Agree and strongly agree	8	28.6	0	-	11	33.3
	Disagree and strongly disagree	6	21.4	1	33.3	9	27.3
	Neutral	14	50.0	2	66.7	13	39.4

Note: Table 3 presents the reasons for vaccine refusal, along with the frequency and percentage of PUBS, PRIVS and PUPBR participants who agreed, disagreed or expressed a neutral stance.

COVID-19, coronavirus disease 2019; PRIVS, private sector; PUBPR, public-private; PUBS, public sector; HCW, healthcare worker.

Table 3 shows that 50% (*n* = 32/64) of participants agreed with concerns regarding the safety of multiple booster doses. Only 14.1% (*n* = 9/64) of participants expressed disagreement in the uncertainty of safety of multiple booster doses, with a higher rate observed in the PRIVS (17.9%, *n* = 5).

Boosters weakening their immune systems were disagreed with by 39.1% (*n* = 25/65) of participants. Notably, this perception was slightly more prevalent in PUBS (39.4%, *n* = 13) than in PRIVS (35.7%, *n* = 10). A total of 40.6% (*n* = 26/64) of participants agreed that they had contracted COVID-19 and acquired antibodies.

Notably, a higher proportion of PUBS participants (51.5%, *n* = 17) agreed that they had developed antibodies as a result of natural COVID-19 infection. Overall, 48.4% (*n* = 31/64) of participants reported neutrality and 35.9% disagreement (*n* = 23/64) with chronic conditions worsening after receiving the vaccine. A minority agreed that their chronic conditions had worsened, with a slightly higher rate seen in PUBS.

About 52.3% (*n* = 34/65) of participants agreed that they experienced side effects after the first dose of vaccine; this was reported in a greater proportion of PRIVS participants (59.3%, *n* = 16). Mild side effects were reported by 36.9% (*n* = 24/65) of participants. Severe adverse events were reported by 4.7% (*n* = 3/64) of participants with PRIVS (7.1%, *n* = 2), recording a higher rate. A total of 42.2% (*n* = 27/64) of participants did not report side effects to HCWs, while 34.4% (*n* = 22/64) recorded receiving medicines for the side effects.

Close to 30% (*n* = 19/64) of participants agreed about the vaccine’s ineffectiveness against new COVID-19 strains, with the highest observed in the PUBS group (33.3%, *n* = 11).

## Reliability and validity

The reliability and validity of the online questionnaire were ensured by designing standardised and clear questions, drawing from validated instruments. The questionnaire was reviewed by public health experts. Pilot testing and refined wording ensured comprehensive coverage of relevant constructs. Anonymity and confidentiality were assured to promote honest responses, and technical functionality was tested across multiple devices. Measures were implemented to prevent duplicate responses and minimise sampling bias, supporting the robustness and credibility of the study findings.

## Discussion

The average vaccine acceptance rate of 92.4% (*n* = 306/331) among HCWs in KZN was notably high, exceeding 90.1% (*n* = 1308) and 89% reported in previous SA studies (Adeniyi et al. 2021; George et al. 2023c). Our study identified that vaccine acceptance across different sectors was associated with demographic factors, educational background, training opportunities and the use of chronic medications.

Conversely, vaccine refusal was associated with perceptions regarding vaccine safety and efficacy, levels of institutional trust, religious beliefs, beliefs in natural immunity and exposure to misinformation.

Approximately half of the participants received the J&J vaccine, mirroring the preferences documented in the Sisonke study and the initial national rollout (NDOH 2021a).

This study’s average booster acceptance rate of 73.7% (*n* = 244) closely aligns with a study from India (Rathinakumar et al. 2024) and is considerably higher than the 56% reported in a recent South African study (George et al. 2023a). This disparity may be because of differences in study populations, timing, regional infection rates and public health messaging (George et al. 2023a). Lower booster rates in this study may be attributed to concerns about safety, potential side effects, beliefs in natural immunity and apprehension regarding the necessity for repeated doses, consistent with findings from previous research (George et al. 2023a).

This study found that age, gender and race emerged as significant factors affecting booster uptake. Our findings indicate that the lower uptake of boosters in individuals aged 50–59 may have been potentially influenced by concerns about side effects and the frequency of additional doses (George et al. 2023a). This outcome diverges from previous global research, which has generally reported higher vaccine acceptance rates among older adults (Biswas et al. 2021).

Despite higher initial vaccine acceptance by PUBS and PRIVS, females were less likely to receive boosters, corroborating findings from another South African study (George et al. 2023a). A global study found that this disparity may be rooted in gender-specific health beliefs and safety concerns (Skjefte et al. 2021).

Racial and sectoral differences were also apparent in booster acceptance between African and white counterparts. This finding suggests that cultural, linguistic or historical trust issues remain barriers to vaccine acceptance (Adhikari, Yeong Cheah & Von Seidlein 2022), emphasising the necessity of tailored, community-specific strategies and communication.

Public-private doctors recorded the highest vaccine uptake, followed by PUBS pharmacists. Possible explanations include pharmacists managing vaccine supply chains and information dissemination (NDOH 2021a). Meanwhile, doctors in both PUBS and PRIVS also noted a slightly higher booster uptake compared with the vaccine. This was consistent with their exposure to emerging COVID-19 variants and evolving SA and global scientific guidance (Biswas et al. 2021; George et al. 2023a). These findings aligned with prior research indicating that vaccine attitudes and behaviours among HCWs are influenced by their workplace context and professional roles (Biswas et al. 2021). Meanwhile, another SA study found that vaccine acceptance was seen to be considerably higher in HCWs with tertiary education, while lower-educated categories of HCWs could have benefited from targeted vaccine education efforts (Adeniyi et al. 2021).

Our study found that the pivotal role of undergraduate education and organisational training in vaccine uptake was evident. The rate of adequate undergraduate vaccine training differed among the sectors, with the highest recorded in PUBPR. This highlighted the need for standardised undergraduate vaccine education programmes. Such standardised curricula would provide essential foundational knowledge and create opportunities to address students’ concerns, reduce vaccine hesitancy and enhance awareness regarding vaccinations (Belingheri et al. 2021). This study identified that limited advanced understanding of vaccines across diverse sectors was a cause for concern, as this may have substantially undermined vaccine confidence, reduced uptake and hindered efforts to effectively combat vaccine-related misinformation (Parisi et al. 2025). Peterson, Lee and Nugent (2022) also noted that the majority of HCWs lack specialised training in immunology and vaccinology; thus, their experience in administering vaccines does not inherently confer an in-depth understanding of vaccine development (Peterson et al. 2022). Limited exposure to the clinical trials and public health initiatives may undermine their confidence in, and perceptions of, vaccine safety and efficacy, potentially contributing to vaccine hesitancy (Peterson et al. 2022). Nevertheless, hesitancy persisted even among some with advanced knowledge, illustrating the complex nature of vaccine acceptance and decision-making (Peterson et al. 2022).

Our study highlighted that PUBS and PRIVS HCWs who refused the vaccine did not promote vaccination to family and friends. This effect was especially marked among PRIVS participants, aligning with the local and international studies that reported that vaccine refusal was consistently linked to a lack of vaccine advocacy (George et al. 2023c; Roberts et al. 2022). This pattern highlighted the extent to which a HCW’s personal vaccination decisions significantly impact their effectiveness as vaccine advocates, potentially influencing overall community vaccine uptake and the spread of positive vaccine information (Belingheri et al. 2021).

Our study concurred with the conclusions of Roberts et al. (2022) identifying the need to change attitudes (Roberts et al. 2022). This can be done by unifying messages, normalising conversations and providing proper training and resources (George et al. 2023c).

Public sector facilities appeared to have more structured or mandatory vaccination training, provided by the NDOH (2021a). While this reflects greater institutional emphasis on staff preparedness and education, our study highlighted that this may not have translated into a higher PUBS vaccine acceptance rate. A blended approach (online and face-to-face) indicates a recognition of diverse learning preferences and the need to use suitable and sustainable training methods for quick, ongoing and tailored training. Our study data indicate a potential association between insufficient training and refusal of vaccines or boosters, suggesting that training may be a factor in promoting vaccine uptake, consistent with another SA study (Wiysonge et al. 2022). Conversely, research conducted in Sierra Leone reported that while HCWs experienced a lack of education, this did not correspond to an unwillingness to receive vaccination (Joseph et al. 2023). As a result of almost one-third of PUBS and PRIVS not being able to ask questions during training, our study reinforces the importance of interactive training sessions, as well as opportunities for questions and critical discussion.

This study noted a higher rate of PRIVS positive COVID-19 tests in comparison to PUBS, which may indicate higher exposure risk in private healthcare settings, and more COVID-19 testing among PRIVS participants. Commonly reported mild or moderate illness in this study aligns with existing evidence that vaccination reduces the severity of COVID-19 outcomes, even when breakthrough infections occur (Huang & Kuan 2022).

Vaccine refusal reasons showed notable differences among PUBS, PRIVS and PUBPR. Both PUBS and PRIVS expressed concerns about vaccine safety, with high proportions agreeing that negative experiences of friends and/or family, a lack of trust in vaccine efficacy and suspicions about manufacturers’ profit motives contributed to their reluctance. These findings echoed in other SA and global studies (Dror et al. 2020; George et al. 2023a). Vaccine accessibility was not identified as a refusal reason in this study, consistent with the findings of another SA study (Naidoo & Taderera 2025). Close to 10% of PUBS and PRIVS participants agreed with the use of traditional medicines for COVID-19 treatment, with a slightly higher rate seen in PRIVS. This perspective diverges from findings from another South African study, which reported that a significant proportion of university staff and students utilised traditional medicines during the nationwide lockdown (Mphekgwana, Makgahela & Mothiba 2021). However, WHO emphasises the importance of an evidence-based approach to traditional medicine, advocating for the establishment of efficacy and safety through rigorous scientific validation (WHO 2025). Additionally, a total of 30% of PUBS and PRIVS in our study cited natural immunity as a reason for vaccine refusal, with a higher rate in PUBS. This is similar to findings of SA and global studies, which underscores natural immunity and a low perceived severity of COVID-19 among these groups (George et al. 2023c; Wong et al. 2023). This study raised concerns about microchips in vaccines (Wong et al. 2023), which were dismissed by the majority of participants in our study. Approximately one-third of study participants expressed distrust in the government, aligning with the primary reasons identified by George et al. 2023c. Religious reasons were cited by about a quarter of PUBS and PRIVS refusing the vaccine, and higher in PRIVS. Similarly, a Malaysian study highlighted the significance of the ingredients and halal status of vaccines on the uptake of vaccines (Wong et al. 2023). This highlights a disparity between religious and traditional leadership and public health programmes. The influence of anti-vaxxers, particularly via social media, remains a significant threat to public health initiatives (Hoare, Mendelson & Frenkel 2022). Effective communication strategies must address misinformation directly and be tailored to the unique challenges of online platforms and resistant groups. The majority of PUBS and PRIVS participants acknowledged encountering negative effects on social media. Social media was consistently identified as a prominent source of adverse information, mirroring global patterns in the dissemination of vaccine misinformation (Skafle et al. 2022). The rapid development of COVID-19 vaccines and uncertainties regarding long-term side effects were particularly influential among PUBS and PRIVS, consistent with research suggesting that perceived scientific uncertainty can undermine vaccine confidence (Dror et al. 2020; George et al. 2023c).

The study found that half of the PUBS and PRIVS participants refusing booster were uncertain about booster safety, mirroring findings both nationally and internationally (Dror et al. 2020; George et al. 2023b). Public sector participants predominantly rejected the belief that booster doses compromise the immune system, reflecting a moderate level of vaccine literacy within this group, potentially linked to their higher rates of training. This is consistent with the findings of Fenta et al. (2023), who concluded that improving health literacy through varied vaccine promotion strategies can increase COVID-19 vaccine acceptance and support informed decision-making, ultimately reducing the impact of the pandemic (Fenta et al. 2023). Almost half of PUBS participants agreed that acquiring natural immunity and antibodies after COVID-19 infection paralleled evidence that perceived natural immunity often diminishes the need for booster uptake (George et al. 2023b; Wong et al. 2023).

While most of the vaccine side effects were mild or moderate, a substantial proportion of the study participants did not report side effects, indicating a need for improved pharmacovigilance education and awareness (South African Health Products Regulatory Authority [SAHPRA] 2023). Concerns about vaccine effectiveness against emerging variants were prominent, with about one-third expressing doubts – consistent with literature highlighting the impact of viral mutations on vaccine efficacy (George et al. 2023a; Ghildiyal et al. 2024).

### Limitations and recommendations

This study has several limitations. Because of the cross-sectional study design, causal relationships cannot be established. Additionally, the use of self-reported data may introduce social desirability bias; for example, HCWs might feel professional pressure to report positive attitudes toward vaccination. The sample may not be representative of all HCWs because of online data collection only, thereby limiting generalisability.

Non-response bias is also possible, as those with strong views on vaccination may have been more likely to participate. The survey format restricted deeper qualitative exploration of attitudes, and the timing of data collection means that findings may not reflect changes in attitudes or policies over time. Additionally, the study did not fully examine the influence of cultural or linguistic diversity on vaccine acceptance.

Based on the findings of this study, several key recommendations are proposed to strengthen vaccine acceptance among PUBS and PRIVS HCWs. First of all, standardised and interactive vaccine education should be integrated into both undergraduate curricula and ongoing professional development to address knowledge gaps and misconceptions. Targeted communication strategies are needed to counteract misinformation (George et al. 2023b), particularly on social media, and should be tailored to address the specific concerns of different demographic groups, including those related to religious beliefs, natural immunity and booster safety. Collaboration with religious and community leaders can help address cultural and faith-based barriers and empower these leaders to be public health advocates supporting HCWs. Additionally, promoting evidence-based guidance on traditional medicine use (WHO 2025) and encouraging HCWs to serve as proactive vaccine advocates within their communities are essential. Institutional support through clear policies, opportunities for open discussion and regular evaluation of intervention effectiveness will further strengthen vaccine confidence and uptake. These approaches collectively aim to reduce hesitancy, build trust and empower PUBS and PRIVS HCWs to drive vaccination programmes more effectively.

## Conclusion

This study concludes that vaccine acceptance and confidence among HCWs are shaped by a complex interplay of workplace environment, demographic factors, training and exposure to information or misinformation.

Notably, PUBPR participants demonstrated the highest rates of vaccine and booster acceptance, suggesting that a dual work environment may provide more practices and educational resources that fostered greater vaccine confidence and uptake. Public sector participants exhibited strong training and showed greater resistance to misinformation. However, PUBS noted firmer belief in acquired immunity and the vaccine’s ineffectiveness against new COVID-19 strains. Moreover, PUBS recorded neutrality towards some conspiracy theories, along with the highest booster refusal rate, reinforcing ongoing uncertainties. In contrast, PRIVS participants reported more chronic medication use and COVID-19 cases, greater uncertainty about vaccine safety and efficacy, and were more influenced by social and religious factors, contributing to higher booster hesitancy.

Across PUBS, PRIVS and PUBPR, attitudes and beliefs were influenced by personal experiences, demographic background, training and social influences.

Addressing the complex determinants of vaccine hesitancy across healthcare sectors necessitates a structured, integrated, and sustainable policy-driven approach reinforced by multidisciplinary stakeholder engagement.

This should commence with the incorporation of vaccine education into undergraduate healthcare curricula, extend to continuous workplace training, and include targeted programmes for religious, community and traditional leaders. The implementation of such comprehensive, proactive strategies is critical for enhancing vaccine confidence, increasing uptake and safeguarding public health, thereby ensuring preparedness for future pandemics.
